# COVID-19 Quarantine Effects on Smoking Behavior and Mental Health of Smoking Adolescents

**DOI:** 10.5152/eurasianjmed.2021.21058

**Published:** 2023-02-01

**Authors:** Demet Taş, Özden Şükran Üneri

**Affiliations:** 1 Department of Pediatrics, Division of Adolescent, Ankara Yıldırım Beyazıt University, Ankara City Hospital, Childrens Hospital, Turkey; 2 Department of Child and Adolescent Psychiatry, Ankara Yıldırım Beyazıt University, Ankara City Hospital, Childrens Hospital, Turkey

**Keywords:** Adolescent, COVID-19, mental health, smoking, quarantine

## Abstract

**Objective:**

The world is struggling with the damage caused by the coronavirus disease 2019 pandemic. Most countries have applied quarantines to combat the spread of coronavirus disease 2019. The aim of this study was to determine the mental health of smoking adolescents and the change in smoking behavior compared to their peers during the coronavirus disease 2019 quarantine.

**Materials and Methods:**

This study was conducted with adolescents registered in the adolescent outpatient clinic with no record of psychiatric illness. The mental health of smoking (n = 50) and non-smoking (n = 121) adolescents was evaluated using the Brief Symptom Inventory. Smoking adolescents have been questioned about the change in smoking behavior since quarantine began.

**Results:**

The rates of depression and hostility symptoms were significantly higher in smoking than in non-smoking adolescents. Male smokers had significantly higher depression and hostility symptoms than male non-smokers. However, no significant difference was observed between the rates of female smokers and non-smokers. It was determined that 54% (27) of the smokers reduced their smoking, while 14% (7) smoked more than before and 3.5% of former smokers stated that they quit smoking during quarantine and these people were included in the non-smoker group.

**Conclusion:**

It is not surprising that the mental health of adolescents was affected by the coronavirus disease 2019 quarantine. Our findings revealed the necessity to closely monitor the mental health of smoking adolescents, especially males smokers. The results of our study suggest that encouraging adolescents who smoke to quit during the coronavirus disease 2019 pandemic may be more effective than before quarantine.

**Main Points**

- Our findings revealed that smoking adolescents showed more psychopathology than their peers during the coronavirus disease 2019 (COVID-19) quarantine.
- We wanted to draw attention to the necessity of closely monitoring the mental health of smoking adolescents, especially males.
- The fact that more than half of the smoking adolescents in our study reduced smoking shows the intention of adolescents in this direction. We consider it significant to encourage and guide smokers to quit smoking during the COVID-19 pandemic.

## Introduction

Recently, countries throughout the world have been struggling with the unexpected emergence of coronavirus disease 2019 (COVID-19). Due to the rapid transmission of the severe acquired respiratory syndrome coronavirus 2 (SARS-CoV-2) virus and the lack of effective treatment, quarantine is regarded as the most effective method of preventing the spread of the disease.^1^ So lockdowns have been declared by many countries due to the COVID-19 outbreak. Closures of restaurants, gyms, entertainment centers, and limitations in social interactions have been shown to negatively affect the psychology of the entire population in a recent review.^2^ Adolescents, who prioritize socialization with peer groups,^3^ may be more negatively affected by the restrictions due to the pandemic. Also, with the closure of schools, adolescents’ routines have been found to be unexpectedly upset due to the pandemic. In this context, the mental health of adolescents has been investigated in some quarantined countries.^4,5^ In addition, COVID-19 is an important source of stress due to its mortality and serious medical morbidity, as well as the routine life difficulties it causes. The destructive effect of the SARS-CoV-2 in the lower respiratory tract determines the prognosis of COVID-19.^6^ A recent review stated that smokers have a higher risk of developing COVID-19 and have a more severe disease.^7^ The harmful effect of smoking on the lower respiratory tract, which is well documented,^8^ can also occur in adolescents,^9^ thus making them more susceptible to the harmful effects of COVID-19. World Health Organization emphasizes that it is the right time to quit smoking during the COVID-19 pandemic to protect health.^10^ According to Green Crescent (non-profit organization combat with addiction) reports, the tendency of adults to quit smoking has increased during the COVID-19 pandemic.^11^ In one study conducted with adults during the first few months of the pandemic, participants indicated their intention to quit smoking; however, in a different study, no significant change in the participant’s attitude toward smoking was observed.^12,13^ Another study found that nearly half of adults who smoked did not change their smoking behavior after learning about the COVID-19 pandemic.^14^ The perceived stress in smoking adolescents may be greater than in their non-smoking peers due to multiple reasons such as quarantine and fear of COVID-19’s mortality and morbidity. To the best of our knowledge, how adolescents’ smoking habits and psychiatric symptoms were affected during the COVID-19 outbreak quarantine has not been studied further. Thus, this current study aims to investigate the psychiatric symptoms and smoking behavior of smoking adolescents in quarantine.

## Materials and Methods

This investigation was a cross-sectional study that was conducted online between April 16 and 26, 2020, which was approximately a month after the start of the quarantine for adolescents. Ethics committee approval was obtained from the Ankara City Hospital Clinical Research Ethics Committee (2020-04).

### Participants and Study Design

Adolescents included in the study were driven from the records between January 2019 and January 2020. Inclusion criteria of smokers and non-smokers are being older than 14, living in the same home with both parents, having no chronic illness, having no relatives or close friends who had positive COVID-19 cases, no other known trauma in the past 3 months, and they had WhatsApp, the cross-platform messaging application, on his or her mobile phone. In our adolescent clinic, psychosocial status is routinely questioned with home, eating, education/employment, activities, drugs, sexuality, suicide (emotions), and safety (HEEADSSS).^15^ As a result of the HEEADSSS query contained in the files, adolescents who smoked at least one cigarette per day and fulfilled the research criteria were included in the study as smokers. Adolescents who came to the adolescent health clinic between the same dates and who did not smoke intermittently or daily were taken as non-smokers. Due to the quarantine period, participants and their parents were called by phone, informed about the research, and their consent was asked (the consent form was also sent to the parents via WhatsApp). Adolescents included in the study were called back between April 16 and 26, 2020. According to the patient registry system, the records of 130 smoking adolescents were accessed. Although several calls were made at different times, 19 parents could not be contacted and 16 were excluded because they did not give consent to the study. The online questionnaire was sent to 95 smoking adolescents to fill in via WhatsApp. The online questionnaire was sent to 200 non-smoking adolescents who met the research criteria.

### Measures

The questionnaire consisted of 2 parts; the first was comprised of questions prepared by the researchers. The last two questions of the first part were asked only to smokers. The common questions for the group of both smokers and non-smokers included age, gender (female, male), the status of continuing formal education (yes, no), school success (according to the grade in the last report for those who go to school: good, medium, bad), family income perception (bad, medium, good), and whether they applied quarantine. The participants were asked whether they have a parent who smokes (yes, no), someone who wanted to quit smoking and whether they knew someone who quit smoking during the quarantine period (family member/other). To determine the possible psychiatric diagnoses of the participants, a question was asked to parents regarding whether a psychologist/psychiatrist had ever seen them and whether they had ever used any medication given to them by a psychiatrist. In addition, it was confirmed by the expert whether the participants had a previous psychiatric diagnosis from their recorded documents. The participants who wanted feedback based on the research results were asked to share their mobile phones.

The questions asked to smokers are:

How many cigarettes per day did you smoke before quarantine? (Options: 5 or less than 5, 5-10, 10-15, 15-20, more than 20). Has your smoking behavior changed since starting quarantine? (Options: I quit smoking, I reduced smoking, not changed, and I increased smoking). The Brief Symptom Inventory (BSI) scale was used to determine the psychiatric symptoms of the adolescents. Brief Symptom Inventory has validity and reliability for Turkish adolescents.^16,17^

### Brief Symptom Inventory

The BSI is a multidimensional screening scale developed to detect psychiatric symptoms that may occur for various reasons. The 53-item self-assessment scale was developed from the 90-item Symptom Check List-90 (SCL-90).^18^ While the BSI was adapted to Turkish by Şahin and Durak,^16,17^ among the 90 items distributed among 9 sub-factors of SCL-90, a total of 53 items with the highest load in each factor were selected, and a short scale of similar structure was obtained. As a result of the analyses, the scale consisted of 5 subscales (with item numbers): anxiety (13), depression (12), negative self (12), somatization (9), and hostility (7). The BSI is a Likert-type scale; the participants were asked to answer the questions by considering the last week. The answers ranged from none (0 points) to very much (4 points). The Global Severity Index (GSI) was calculated by adding up all the scores and dividing the total number of the scale by 53; results above 1.0 (cut-off point) were considered psychopathology. The GSI value of less than 1.0 indicates that these symptoms were not at the psychopathological level. The scoring of the sub-categories was calculated by dividing the total of the relevant scores by the number of relevant questions in that category. The cut-off point for psychopathology related to the sub-category was considered 1.0. As a result of the analyses during the adaptation of the scale, the Cronbach’s alpha internal consistency coefficients were found to be between 0.96 and 0.95, and the coefficients obtained for the subscales were between 0.75 and 0.88.

### Study Analysis

**Figure 1.**
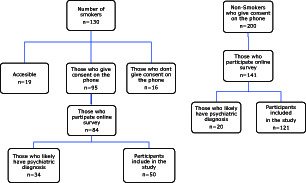
Flowchart of participant adolescents.

Of the potential participants, 64% (84) of the smokers and 70% (141) of the non-smokers participated in the study. Totally, 40% (34) of the smokers and 14.1% (20) of the non-smokers were excluded from the study because they may have a psychiatric illness. After excluding those with a possible psychiatric diagnosis from the study, analyses were made with 50 smokers and 121 non-smokers. A flowchart of participating adolescents is given in Figure 1. It was determined that 3 (3.5%) of 84 smoking adolescents quit smoking during the quarantine period. Those were included in the non-smoker group since they did not have psychiatric comorbidity and did not smoke during the analysis phase of the study. IBM Statistical Package for the Social Sciences for Windows Version 23.0 was used for the statistical analysis (SPSS, IBM, Statistics for Windows [Computer Program]. Version 23.0. 2015.). Categorical variables were shown by frequency and percentage. Numerical variables were summarized as mean standard deviation. The chi-square test and the Fisher-Freeman–Halton test were used to examine whether there was a difference between the groups in terms of the variables. The Mann–Whitney *U* test was used to evaluate the differences in numerical variables between the 2 groups since parametric test hypotheses were not provided. The significance level was determined as *P* ≤ .05.

## Results

The mean age of the participants (n = 171) was 16.80 ± 1.46; the mean age of the non-smokers (n = 121) and smokers (n = 50) was 16.45 ± 1.45 and 17.64 ± 1.12, respectively.

The age difference between smokers and non-smokers was found to be significant (*P* < .001). The rates of the smoker and non-smoker males were 62% and 37%. The gender difference between smokers and non-smokers was found to be significant (*P* < .001).

When other demographic data of smoking and non-smoking adolescents are compared, the difference between dropout rates, school success, family members smoking, adaptation to quarantine, the rate of family members who quit smoking during quarantine, and the rates of those who want to receive feedback according to the scale results were found to be significantly different. These differences and statistical significance values are given in Table 1.

In smoking adolescents, the rate of those smoking 5 or less in a day is 66% (33), while it is 22% (11) for those smoking between 10 and 15. In addition, the rates of adolescents smoking between 15 and 20 and more than 20 in a day were found to be 8% (4) and 4% (2), respectively. None of the smokers in our study group reported that they smoke an average of 5-10 cigarettes per day.

During quarantine, it was determined that 54% (27) of the smokers reduced smoking, 32% (16) smoked the same amount as before, and 14% (7) smoked more than before.

For smokers who dropped out of school and those who did not, there was no significant difference in terms of the rate of psychopathology according to BSI scores (*P* = .083). There was no significant difference in terms of the rate of psychopathology according to BSI scores for those who smoked the same amount of cigarettes as before the pandemic and who increased or decreased smoking (*P* = 1.000).

There was no relationship between having a psychiatric symptom at a psychopathological level and the number of cigarettes smoked per day (*P* = .530).

Among all the participants who wanted to get feedback, the rate of those whose BSI somatization score was at a psychopathological level was 37.8%, while the rate of those who did not want to receive feedback was 15.7%, and the difference was significant (*P* = .003).

Depression and hostility rates were found to be significantly higher in smokers compared to non-smokers (*P* = .016, *P* = .007). There was no significant difference between the rates of psychiatric symptoms of male smokers and female smokers (*P* = .276). When evaluated according to gender, depression, and hostility, the rates were found to be significantly higher in male smokers compared to male non-smokers (*P* = .001, *P = *.002). However, there was no significant difference between the rates of psychiatric symptoms of females (*P* = .075). Anxiety and depression rates were found to be significantly higher in female non-smokers compared to male non-smokers (*P* = .038, *P *< .001). The rate of those who were above the cut-off points of psychopathology for BSI-GSI and their subscales are shown in Tables 2, 3, and 4.

## Discussion

Tobacco and tobacco products are strongly addictive substances that are widely used. There is limited medical literature on how pandemic features affect addiction-related processes. In our study, during the COVID-19 pandemic, psychiatric complaints were screened using BSI in smoking and non-smoking adolescents. The effect of the pandemic and quarantine on smoking behavior was questioned in the smoking group. Smoking adolescents were found to show higher rates of psychiatric symptoms during quarantine than non-smokers. Depressive and hostility symptoms of smokers were both significantly higher than for non-smokers. Since it is stated in the literature^19^ that people with psychiatric illnesses smoke more, smokers with a diagnosis or suspicion of psychiatric illness were excluded from the study. There is a bidirectional relationship between smoking and psychiatric diagnosis. Previous studies examining the relationship between smoking and depression symptoms have focused on multiple mechanisms to explain this relationship. The first is that people with depressive complaints tend to smoke to reduce these symptoms (self-medication theory).^20^ The second theory proposed as an alternative to the self-medication theory is that smoking increases the vulnerability to stress by creating a neuro-cyclical effect, which increases the susceptibility to depressive symptoms.^21^ In the face of compulsory behavior, young people may experience conflicts of authority, ups, and downs in their psychology and demonstrate some behaviors such as depression and hostility.^22^ In our study, smoking adolescents were found to have difficulty in complying with quarantine. This could be interpreted to mean that smoking increases the susceptibility to quarantine-related stress factors since those with possible psychiatric diagnoses were excluded from our study. It may also have caused stress due to the difficulty of secretly smoking from the family during the quarantine period for some adolescents.

When smokers were evaluated according to their gender, there was no significant difference between the rate of smoking females and males with psychiatric symptoms at the psychopathological level. Although stress-related depression is more common in females than male adolescents,^23^ our study results show that male smokers were affected as much by stress caused by COVID-19 and quarantine as female smokers. In our study, the majority of those who smoked 10 or more cigarettes a day were male adolescents. Therefore, risk perception related to COVID-19 may be felt more in male adolescents, which may have increased their psychiatric symptoms.

In our study, the symptoms of depression and hostility were found to be higher in male smokers than they were in male non-smokers. However, the psychiatric symptom rates of smoker and non-smoker girls were similar, although the depressive symptom rates were high in both groups. This suggests that depressive symptoms due to COVID-19 and quarantine-related stress may have increased in non-smoker females. In a recent study in China, it was noted that adolescents had increased depressive symptoms during the COVID-19 pandemic, but females were more affected.^4^ According to studies, females react with more emotional responses to life stressors due to the effects of their genetics and hormones. It is also stated that the frequency of depressive symptoms in female adolescents is twice that of male adolescents.^24^

The depressive symptoms of the non-smoker adolescents in our study were found to be 52.1%, which is similar to the depressive symptoms of high school adolescents (45%-59.9%) in a study conducted during the COVID-19 pandemic in China.^4^ The prevalence of depressive disorder diagnosed clinically in adolescents aged 15-18 is between 17% and 25%, which is higher than for other age groups.^25^ However, this rate may be higher in screening with self-reporting. In a study conducted with high school students, it was found that approximately 40% of the adolescents showed depressive symptoms according to a self-reported scale.^26^

According to our study, more than half of the smokers stated that they smoked less before than they did during quarantine. In previous studies, the rate of smoking increased after a natural disaster and after a terrorist attack.^27,28^ However, the situation caused by the COVID-19 pandemic is not similar to previous stressful life events. Coronavirus disease 2019 causes serious morbidity and mortality, and its prognosis is determined by its destruction in the lungs.^6^ The morbidity of smoking in the lungs is well known.^29^ The perception of harm caused by the SARS-CoV-2 virus may have increased the perceived stress. In a recent short report, it was found that, after learning of the COVID-19 pandemic, almost half of the smoker adults did not change their smoking behavior, a quarter of them reduced their smoking and approximately one-third increased it.^14^ The results of our study suggest that the effect of the COVID-19 pandemic on smoking behavior in adolescents may differ from that of adults. Our study findings demonstrate that smoking will decrease in the adolescent age group in a condition where smoking is perceived as a risk factor. The somatization symptoms of the participants who wanted to get feedback on psychiatric symptoms were found to be higher. This can be interpreted as a help-seeking behavior. (20). 

Previous researches show that smoking is associated with dropping out of school, the presence of smokers in family members, and low school achievement.^30,31^ Similarly, we found that school dropout and low school success rates were higher in smokers than they were in non-smokers. In our study, similar to the literature, most of the participants who smoked were male. It was also found that at least one parent of the smoking adolescents was a smoker.

In conclusion, the pandemic caused by COVID-19 may have adversely affected the mental health of male smokers in the adolescent age group more than their non-smoker peers. Since this study was conducted with adolescents who visited the adolescent outpatient clinic for any reason, the results of our study may not represent all of the smoking adolescents in the community. This finding suggests that it would be beneficial to provide psychological support to all smoker adolescents, especially males, since the pandemic process affects many aspects of their daily life, even if they do not have psychiatric comorbidity. The fact that more than half of the smoking adolescents in our study reduced smoking shows the intention of adolescents in this direction. It would be beneficial for physicians to question the psychosocial status of smoking adolescents and to guide them toward quitting smoking both during and after the COVID-19 pandemic.

### Limitations

Smokers frequently have comorbid psychiatric diseases.^19^ For this reason, it can be thought that psychiatric symptoms seen in smoking participants may already be expected. Therefore, participants who had a possible psychiatric diagnosis or symptom at any time or who used medical drugs, or had previously used those were not included in the study. Moreover, smoking adolescents were not asked about previous smoking cessation attempts. It may be considered that some of those who reduced their smoking had previously intended to quit. If the psychiatric symptoms of the participants had been evaluated in the clinic, the rates determined would have probably been less. For this reason, the psychiatric symptoms of the participants were interpreted for the evaluations other than comparison, considering the previous studies conducted with the self-report scale.^26^ The effect of the restrictions of parents on the smoking of adolescents has not been evaluated. Although it was not one of our research purposes, adolescents who smoke without their parents’ knowledge may also be affected by this situation. Another limitation of our study is that our results only reflect the situation in the first month of quarantine. It is thought that future studies, without our research limitations and with large-sample and longitudinal investigations, will be beneficial in terms of increasing our knowledge on the subject and planning the requisite protective studies.

## Figures and Tables

**Table 1.** Demographic Characteristics of the Participants

Age ± SD (median) (min-max)		16.80 ± 1.46 (17) (14-19)	17.64 ± 1.12 (18) (14-19)	16.45 ± 1.45 (16) (14-19)	<.001^*^
		All Participantsn (%)	**Smokers** n (%)	**Non-smokers** n (%)	***P***
Gender	Female	60.2 (103)	38 (19)	69.4 (84)	<.001
	Male	39.8 (68)	62 (31)	30.6 (37)	
Current status of education	Continue formal education	82.5 (141)	70 (35)	91.7 (111)	<.001
	Drop out of School	17.5 (30)	30 (20)	8.3 (10)	
School success	Good	65.5 (112)	54(27)^a^	70.2 (85)^a^	<.001
	Medium	24 (41)	22(11)^a^	24.8 (30)^b^	
	Bad	4.7 (8)	4 (2)^b^	5 (6)^b^	
Family income status	High	35.1 (60)	26 (13)	38.8(47)	.204
	Medium	60.8 (104)	68 (34)	57.9 (70)	
	Low	4.1 (7)	6 (3)	3.3 (4)	
Quarantine status	Quarantined	93.6 (160)	82 (41)	98.3 (119)	<.001
	Not quarantined	6.4 (11)	18 (9)	1.7 (2)	
Smoking parents	Yes	59.6 (102)	72 (36)	54.5 (66)	.034
	No	40.4 (69)	28 (14)	45.5 (55)	
Wanted to quit smoking	Herself/himself	4.6 (8)	16 (8)^c^	0	.034
	At least a family member	47.3 (81)	34 (17)^d^	52.9 (64)	
	Friends/others	4.6 (8)	0	6.6 (8)	
	None	43.2 (74)	50 (25)	40.5 (49)	
Quit smoking during quarantine	Herself/himself	1.8 (3)		3	.313
	At least a family member	5.8 (10)	10 (5)	4.1 (5)	
	Friends/others	8.2 (14)	10 (5)	7.4 (9)	
	None	84.2 (144)	80 (40)	86 (104)	
Who wants to get feedback	Yes	21.6 (37)	36 (18)	15.7 (19)	.003
	No	78.4 (134)	64 (32)	84.3 (102)	

In binary comparisons involving more than 4 cells related to the variables in the smokers and non-smokers column, there is a significant difference between the “a-b” and “c-d.”

^*^ *P*-value for Mann–Whitney *U* test.

SD, standard deviation.

**Table 2.** The Rate of BSI Scores at Psychopathological Levels in Smoking and Non-smoking Adolescents

	Smokers (n = 50), %	Non-smokers (n = 121), %	*P *
Anxiety	36	32.2	.634
Depression	72	52.1	.016
Negative self	44	37.2	.407
Somatization	26	18.2	.249
Hostility	72	49.6	.007
BSI-GSI	50	31.4	.022

BSI, Brief Symptom Inventory; GSI, Global Severity Index.

**Table 3.** The Rate of BSI Scores at Psychopathological Levels in Smoking and Non-smoking Adolescents by Gender

	Smokers			Non-smokers		
	Females (n = 19), %	Males (n = 31) ,%	*P *	Females (n = 84), %	Males (n = 37), %	*P *
Anxiety	36.8	35.5	.923	38.1	18.9	.038
Depression	78.9	67.7	.392	63.1	27	<.001
Negative self	47.4	41.9	.707	40.5	29.7	.260
Somatization	26.3	25.8	.968	21.4	10.8	.163
Hostility	63.2	77.4	.276	53.6	40.5	.187
BSI-GSI	63.2	41.9	.276	39.3	13.5	.005

BSI, Brief Symptom Inventory; GSI, Global Severity Index.

**Table 4. t4-eajm-55-1-14:** The Rate of BSI Scores at the Psychopathological Levels in Adolescents According to Gender, Smoking, or Not

	Female			Male		
	Smokers (n = 19), %	Non-smokers (n = 84), %	*P *	Smokers (n = 31), %	Non-smokers (n = 37), %	*P *
Anxiety	36.8	38.1	.919	35.5	18.9	.123
Depression	78.9	63.1	.284	67.7	27	.001
Negative self	47.4	40.5	.614	41.9	29.7	.294
Somatization	26.3	21.4	.424	25.8	10.8	.106
Hostility	63.2	53.6	.610	77.4	40.5	.002
BSI-GSI	63.2	39.3	.075	13.5	41.9	.008

BSI, Brief Symptom Inventory; GSI, Global Severity Index.
